# Correction: Stepwise visualization of membrane pore formation by suilysin, a bacterial cholesterol-dependent cytolysin

**DOI:** 10.7554/eLife.06740

**Published:** 2015-02-11

**Authors:** Carl Leung, Natalya V Dudkina, Natalya Lukoyanova, Adrian W Hodel, Irene Farabella, Arun P Pandurangan, Nasrin Jahan, Mafalda Pires Damaso, Dino Osmanović, Cyril F Reboul, Michelle A Dunstone, Peter W Andrew, Rana Lonnen, Maya Topf, Helen R Saibil, Bart W Hoogenboom

Leung C, Dudkina NV, Lukoyanova N, Hodel AW, Farabella I, Pandurangan AP, Jahan N, Pires Damaso M, Osmanović D, Reboul CF, Dunstone MA, Andrew PW, Lonnen R, Topf M, Saibil HR, Hoogenboom BW. 2014. Stepwise visualization of membrane pore formation by suilysin, a bacteria cholesterol-dependent cytolysin. *eLife* **3**:e04247. doi: http://dx.doi.org/10.7554/eLife.04247

Published 2 December 2014

In the published article there was an error in the schematic representation of suilysin prepore proteins in Figure 6, showing these proteins in the wrong orientation with respect to the membrane.

The figure has been adjusted to show the correct orientation, consistent with the scientific representation in Figure 1.

The corrected figure is shown here:

**Figure 1:**
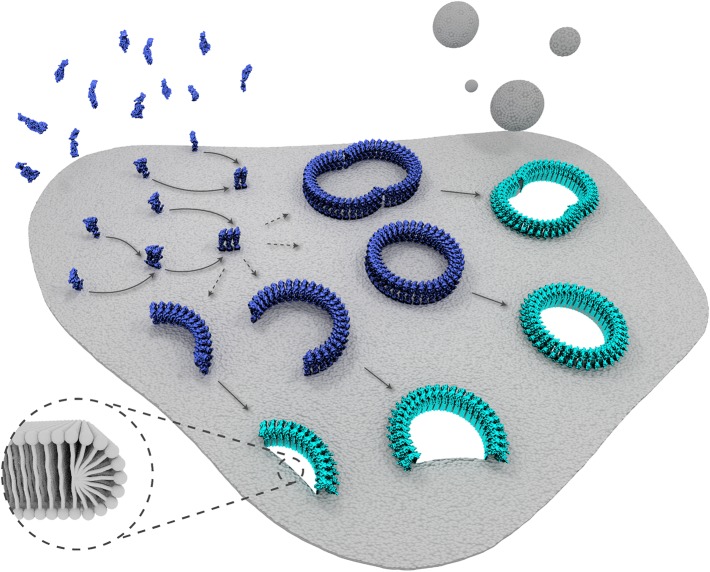
The originally published figure is also shown here for reference:

**Figure 2:**
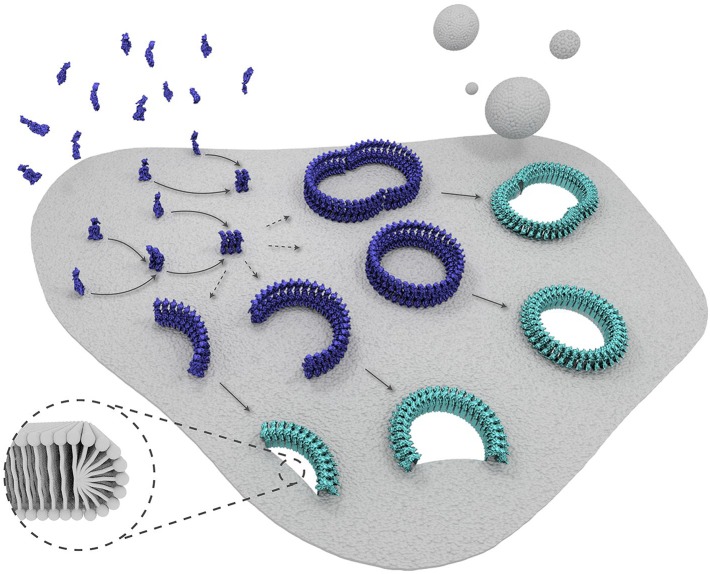
The article has been corrected accordingly.

